# Topical Nasal Anesthesia in Flexible Bronchoscopy – A Cross-Over Comparison between Two Devices

**DOI:** 10.1371/journal.pone.0150905

**Published:** 2016-03-15

**Authors:** Thomas Fuehner, Jan Fuge, Meike Jungen, Anna Buck, Hendrik Suhling, Tobias Welte, Jens Gottlieb, Mark Greer

**Affiliations:** Department of Internal Medicine, Hannover Medical School, Biomedical Research in Endstage and Obstructive Lung Disease Hannover (BREATH), Member of the German Center for Lung Research (DZL); University of California Los Angeles, UNITED STATES

## Abstract

**Introduction:**

Topical airway anesthesia is known to improve tolerance and patient satisfaction during flexible bronchoscopy (FB). Lidocaine is commonly used, delivered as an atomized spray. The current study assesses safety and patient satisfaction for nasal anesthesia of a new atomization device during outpatient bronchoscopy in lung transplant recipients.

**Methods:**

Using a prospective, non-blinded, cross-over design, patients enrolled between 01-10-2014 and 24-11-2014 received 2% lidocaine using the standard reusable nasal atomizer (CRNA). Those enrolled between 25-11-2014 and 30-01-2015, received a disposable intranasal mucosal atomization device (DIMAD). After each procedure, the treating physician, their assistant and the patient independently rated side-effects and satisfaction, basing their responses on visual analogue scales (VAS). At their next scheduled bronchoscopy during the study period, patients then received the alternative atomizer. Written consent was obtained prior to the first bronchoscopy, and the study approved by the institutional ethics committee.

**Results:**

Of the 252 patients enrolled between 01-10-2014 and 30-01-2015, 80 (32%) received both atomizers. Physicians reported better efficacy (p = 0.001) and fewer side effects (p< = 0.001) for DIMAD in patients exposed to both procedures. Among patients with one visit, physicians and their assistants reported improved efficacy (p = 0.018, p = 0.002) and fewer side effects (p< = 0.001, p = 0.029) for the disposable atomizer, whereas patients reported no difference in efficacy or side effects (p = 0.72 and p = 0.20). No severe adverse events were noted. The cost of the reusable device was 4.08€ per procedure, compared to 3.70€ for the disposable device.

**Discussion:**

Topical nasal anesthesia via a disposable intranasal mucosal atomization device (DIMAD) offers comparable safety and patient comfort, compared to conventional reusable nasal atomizers (CRNA) in lung transplant recipients. Procedural costs were reduced by 0.34€ per procedure.

**Trial Registration:**

clinicaltrials.gov NCT02237651

## Introduction

Flexible bronchoscopy (FB) represents the gold standard for diagnosing and treating airway diseases [1]. It offers a proven safety record, good reported patient comfort and avoids the need for general anesthesia [2, 3]. In recent years, nasal intubation has become the preferred method in most centers [4, 5, 6]. Patients however, usually perceive the procedure as potentially uncomfortable, expressing fear of pain, difficulty breathing, nasopharyngeal irritation, or other complications [7]. Light sedation with intravenous midazolam or hydrocodone has been shown to reduce cough, particularly when invasive diagnostic procedures are performed [4, 8, 9]. Some centers however, avoid routine sedation for basic diagnostic bronchoscopy involving bronchoalveolar lavage (BAL) and even transbronchial biopsy (TBB) [10].

Topical airway anesthesia is key to ensuring patient comfort during FB [11, 12]. Many centers topically administer lidocaine due to its rapid onset, short duration of action and low toxicity profile [13]. Current guidelines recommend using 2% lidocaine gel intranasal and 1% lidocaine within airways [4, 9]. Recently Kaur *et al*. confirmed non-superiority of 2% versus 1% lidocaine as topical endobronchial anesthesia, concluding that 1% lidocaine should be preferred [14].

Various modes of administration for topical anesthesia have been reported, including soaked swabs, direct instillation, aerosol spray, nebulization, transcricoid or transtracheal injection, local nerve block, or the “spray-as-you-go technique” (through the working channel of the bronchoscope) [9, 15, 16, 17, 18]. Interestingly, placebo-controlled trials comparing use of nebulized lidocaine versus nebulized saline, in combination with topical lidocaine in the nasopharynx, vocal cords and airways revealed no difference with regard to coughing or patient discomfort [19, 20].

To our knowledge, little data exists regarding applicator devices for administering lidocaine prior to transnasal FB. We performed a prospective study to compare a new disposable Intranasal Mucosal Atomization Device (DIMAD) with a conventional reusable nasal Atomizer (CRNA) for nasal anesthesia for FB in LTx patients.

## Material and Methods

### Study design and patient collective

A single centre, prospective open label cross-over study was performed. Patients were recruited from those attending for bronchoscopy at our lung transplant outpatient clinic between October 2014 and January 2015. All patients were accustomed to our standard bronchoscopy protocol, where transnasal FB with topical anaesthesia is routinely performed without sedation. All patients provided written informed consent. The study was approved by the Internal Review Board of the Hannover Medical School (No. 2354–2014) and registered under clinicaltrials.gov (NCT02237651).

**Fig 1:**
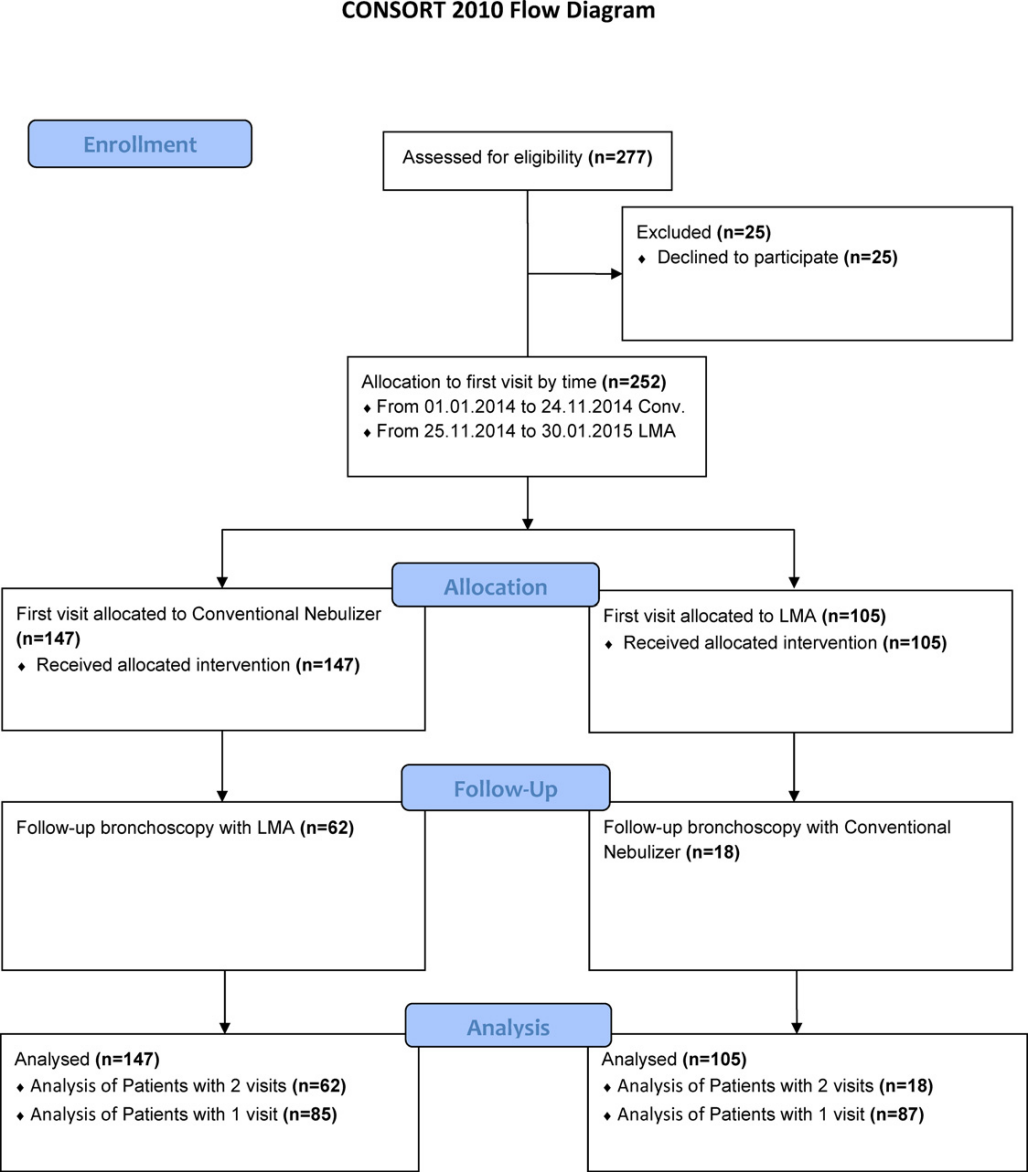
Flow-chart.

**Fig 2:**
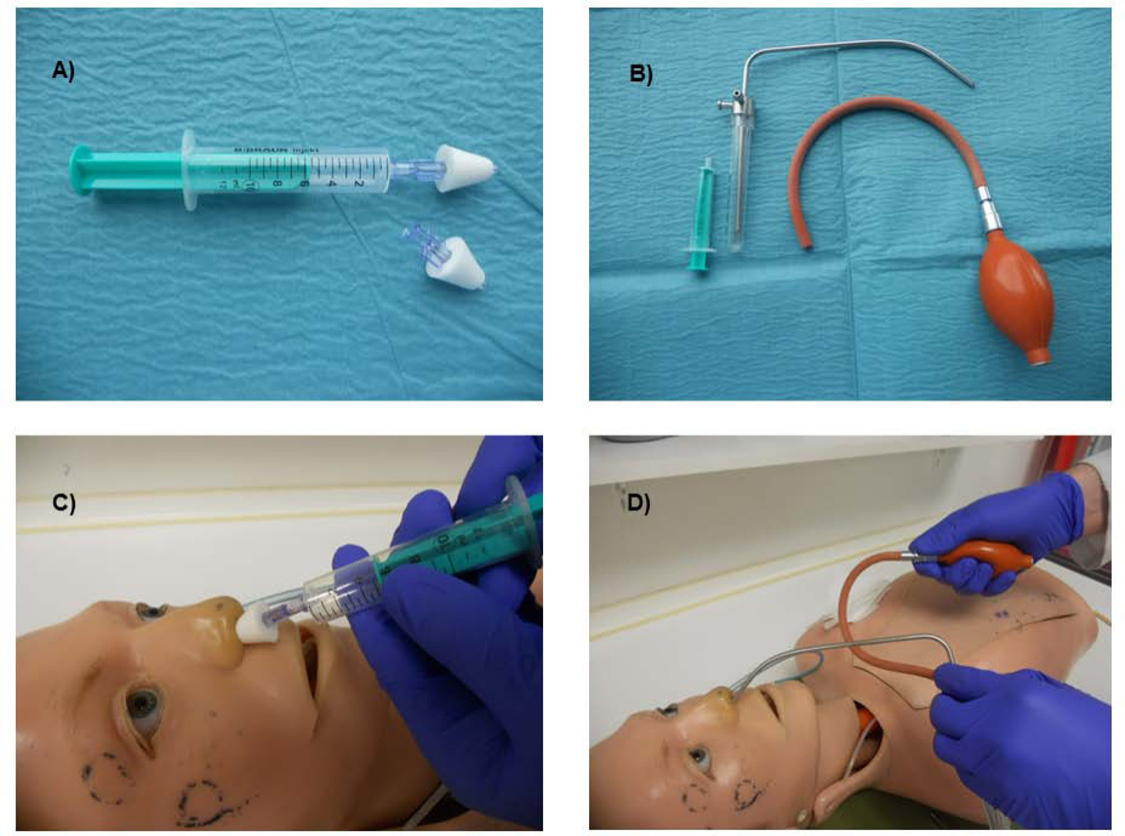
Atomizer Devices.

Between 01-10-2014 and 24-11-2014 (Flowchart Fig 1), participants initially received topical anesthesia using the conventional reusable nasal Atomizer (CRNA) (Storz, Tuttlingen, Germany, = conventional group) (Fig 2). Patients enrolled between 25-11-14 and 30-01-2015, first received nasal anesthesia via the Disposable Intranasal Mucosal Atomization Device (DIMAD), single-use product (LMA® MAD Nasal™, Teleflex medical, Kernen Germany disposable group) (Fig 2). Participants returning for scheduled repeat bronchoscopy within the study period, then received the alternate device. Following each procedure, the patient, attending physician and their assistant independently answered questionnaires rating perceived satisfaction and side effects.

### Inclusion and exclusion criteria

Participants were all aged ≥18 years, and had previously undergone single, double or heart-lung transplantation. All had regularly participated in our post-transplantation surveillance program. Patients were excluded if they required sedation prior to bronchoscopy, if transnasal intubation was known to be impossible or if they were oxygen dependent. Other issues, such as illiteracy, limited German language skills, multi- or pan-resistant colonisation requiring isolation that limited patient communication or computer handling were considered as additional exclusion criteria.

### Endpoint

The primary endpoints of the study were patient satisfaction and perceived efficacy of topical anesthesia, rated using a visual analogue scale. Secondary endpoints included safety, required lidocaine dose, comparison of efficacy between applicator, the need for rescue anesthesia, time local anesthesia to investigation, time of nose passage, examination duration and use of rescue anesthesia.

### Bronchoscopy procedure

Bronchoscopy was performed as described previously by Rademacher *et al*. [10]. All procedures were performed using flexible videoscopes (Olympus, Tokyo, Japan; Types P180, Q180/190, TQ180, T180 and TH 190) with outer diameters of 4.9 to 6.2 mm. Oxygen saturation and heart rate were monitored throughout the procedure. Independent of the atomizer system used, up to 6ml 2% lidocaine was administered intra-nasally, followed by 4 ml 2% Lidocaine gel immediately prior to bronchoscopy. Upon insertion of the bronchoscope, up to 12ml 2% lidocaine was administered via the working channel on a spray-as you go basis, targeting the vocal cords, trachea and proximal bronchial system. If required, 10% Lidocaine spray was available as rescue therapy for the nasopharynx, with the required dose being recorded.

Following inspection of the airways to sub-segmental level, bronchoalveolar lavage (BAL) usually was performed (with 6 × 20 ml aliquots of 0.9% saline instilled and gently aspirated from a sub-segmental bronchus in the region of interest). Transbronchial biopsy (TBB) was performed without fluoroscopy in a single lobe, involving sampling from several segments, aiming to obtain five tissue samples as recommended [21]. Bleeding post-biopsy was managed by instilling aliquots of ice-cold saline or epinephrine (1:20.000) into the bleeding source whilst applying wedged compression with the bronchoscope.

### Questionnaires

Participating patients answered a structured 13-item questionnaire on a tablet-PC in the bronchoscopy suite. Efficacy and side-effects were rated on a visual analogue scale (0 –worst, 10—best). Questionnaires were completed using FileMaker Go (v. 13, FileMaker Inc., USA) installed on Apple iPads (Apple Inc. USA), with data being transferred via WiFi in real time to a study database in FileMaker Pro 12 Server hosted on the local intranet. In addition, both assistants and physicians independently rated efficacy and side effects on a visual analogue scale after the examination via tablet-PC (please see: Supporting Information files; S1 Questionnaire and S2 Questionnaire for English version).

### Cost calculation

Costs were calculated by measuring time of preparation and application and cost of consumables and reprocessing.

### Statistical analysis

The sample size calculation was for two independent groups (with changing allocation). Assuming an effect size of 0.5 we calculated a sample size of 105 visits per group while having set the alpha error probability to 0.05 and the 1-ß error probability (Power) to 0.95. This estimation was derived from previous work [10].

The IBM SPSS Statistics 22.0 (IBM Corp, Armonk, NY, USA) and STATA 13.0 (StataCorp LP, College Station, Texas, USA) statistical software were used to analyze the data. All continuous variables are presented as means with standard deviation (SD) or median with inter-quartile ranges (25% and 75%). Variables were compared between the groups using student’s t-test or non-parametric testing (Mann Whitney U) in cases of non-normal distribution. Categorical variables were compared between the groups using the chi-square test. All reported P values are two-sided and the level of significance was set at p < 0.05.

## Results

In total 252 patients were enrolled. Details of patient allocation, follow-up and inclusion in analysis are summarized in Fig 1. Eighty out of 252 patients (32%) were subjected to both devices during the period of observation. A further 87 patients (34%) were treated with the disposable atomizer only, the remaining 85 patients (33%) received only the conventional atomizer.

**Fig 3:**
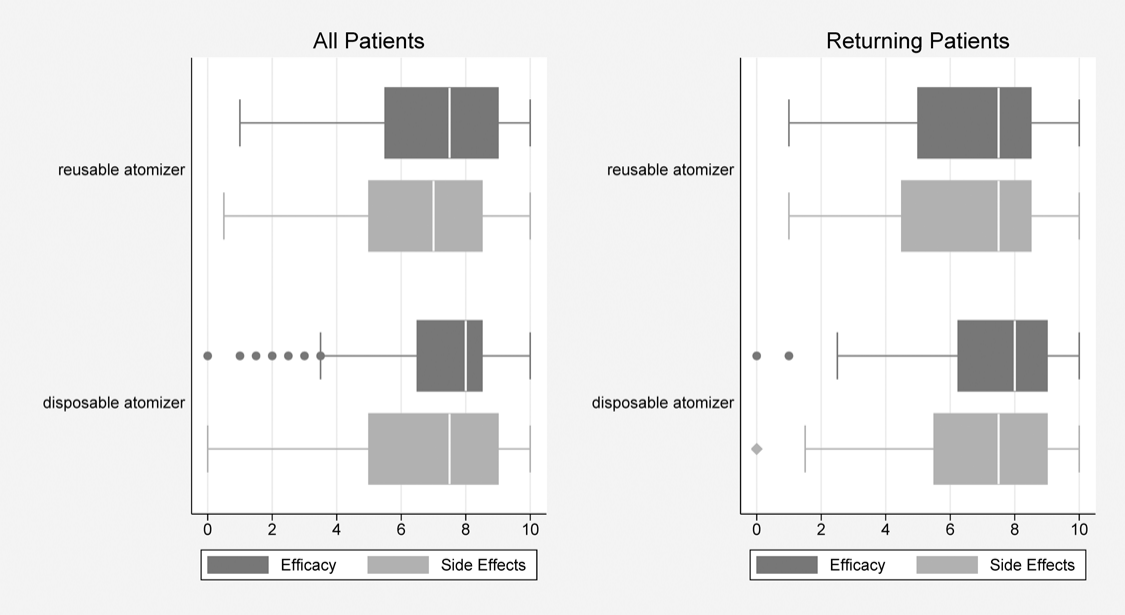
Results.

Nasal insertion of the bronchoscope was successful in all patients. The questionnaire completion rate was 100% and all were included in the analysis (Fig 3).

Study demographics are summarized in Table 1. Similar patient characteristics were observed in all groups. All but 4 patients had undergone bilateral lung transplantation. One third of patients had known chronic lung allograft dysfunction. All patients had previously undergone numerous bronchoscopies, with no significant differences between groups. Physician experience with bronchoscopy varied, with no significant inter-group differences. Bronchoscopy duration varied between 12 and 15 min. The rates of bronchoalveolar lavage were 90 *vs*. 98% and transbronchial biopsy 39 *vs*. 55% respectively. The mean lidocaine dose was 5.8 mg/kg (5.4–6.2 95%CI) vs. 6.2 mg/kg (5.8–6.6 95%CI) total and 140 mg (132–148 95% CI) vs. 146 mg (138–154 95%CI) nasal.

**Table 1:** Demographics.

Characteristics	Patient with one visit (N = 172)	Patients with two visits (N = 80)	All Patients (N = 252)
Age—**Median (IQR)**	53 (43–60)	51 (37.5–58.5)	52 (41–59.5)
Sex—**n (%)**			
** - Male**	96 (56)	46 (58)	142 (56)
** - Female**	76 (44)	34 (43)	110 (44)
Tx Type—**n (%)**			
** - Bilateral**	169 (98)	79 (99)	248 (98)
** - Unilateral**	1 (1)	0 (0)	1 (0)
** - heart lung**	2 (1)	1 (1)	3 (1)
Age at last Transplantation—**Median (IQR)**	50 (39–58)	50 (36–57)	50 (38.5–57)
Diagnosis—**n (%)**			
** - cystic fibrosis**	45 (26)	18 (23)	63 (25)
** - pulmonary fibrosis**	46 (27)	31 (39)	77 (31)
** - emphysema**	55 (32)	17 (21)	72 (29)
** - Pulmonary hypertension**	6 (4)	2 (3)	8 (3
** - Other**	20 (12)	12 (15)	32 (13)
BOS Stage—**n (%)**			
** - no baseline value available**	10 (6)	2 (3)	12 (5)
** - BOS 0/0p**	115 (67)	57 (71)	172 (68)
** - BOS 1**	26 (15)	10 (13)	36 (14)
** - BOS 2**	12 (7)	6 (8)	18 (7)
** - BOS 3**	9 (5)	5 (6)	14 (6)
Years after transplant—**Median (IQR)**	1.7 (0.9–3.1)	0.8 (0.5–2.0)	1.4 (0.7–2.7)
Number of previous Bronchoscopies—**Median (IQR)**	13 (10–19)	15 (11–27.5)	14 (10–20.5)

Data as of 11 Feb. 2015; All numeric variables are shown as median with interquartile range (IQR); All categorial variables are shown as N (%);

### Endpoint outcomes

Patient satisfaction for both devices was high (68–74 on a visual analogue scale with 100 being the maximal satisfaction), with physicians and assistants reporting similar findings. Among single-visit patients, no difference in satisfaction between devices was evident (Table 2). In the sub-group of single-visit patients however, the assistants rated the disposable atomizer´s efficacy as significantly better (p = 0.002). Physicians rated the disposable device significantly better among patients subjected to both devices (p = 0.018). Considering all patients who were treated with the disposable atomizer (DIMAD), a significant majority preferred this device (“improved efficacy”, “improved side effects”; p<0.001, Tables 2 and 3). Use of rescue medication was similar in both groups (First visit: p = 0.41, Returning visit: p = 0.11). Side-effects of topical nasal lidocaine were acceptable, 65% (60–68 95% CI) vs 71% (68–75 95% CI) on visual analogue scale with both devices. Side effects with the disposable atomizer were rated similar by patients (p = 0.202), and lower by assistants (p = 0.029) and physicians (p<0.001) among single attendees (Table 2). Only physicians rated improved satisfaction of side effects in local anaesthesia with DIMAD for patients attending twice (p<0.001) (Table 3). No severe adverse events occurred, with no deaths or attributable hospitalizations ensuing. Epistaxis was reported in 6 patients, shared between devices. Two patients suffered prolonged but reversible desaturations during bronchoscopy (Table 4). The proportion of patients with no anesthesia-related side effects tended to be lower in the disposable group, although none achieved significance. The most frequent side effects were pain (28–43%), burning sensation (33–53%) and the unpleasant taste (71–75%) of the spray.

**Table 2:** Results for patients with first visit (Primary Endpoints).

Characteristics	Conventional Nebulizer (N = 147)	DIMAD, LMA®(N = 105)	p-value
Patient satisfaction VAS			
** - Efficacy**	7.27 ±2.2	7.37 ±2.1	p = 0.725
** - Side Effects**	7.03 ± 2.2	6.64 ±2.6	p = 0.202
Physician satisfaction VAS			
** - Efficacy**	7.24 ±2.4	7.93 ±2.1	p = 0.018
** - Side Effects**	8.45 ±2.1	6.82 ±2.8	p < = 0.001
Assistant satisfaction VAS			
** - Efficacy**	6.22 ±2.5	7.17 ±2.1	p = 0.002
** - Side Effects**	6.46 ±2.5	7.18 ±2.6	p = 0.029
Improved efficacy in comparison to previous bronchoscopiesw—**n (%)**	33 (22)	60 (57)	p < = 0.001*
Improved side effects in comparison to previous bronchoscopies—n (%)	29 (20)	60 (57)	p < = 0.001*
Time local anesthesia to investigation, min	2:07 ±0:30	2:11 ±2:14	p = 0.651
Time of nose passage, min	0:23 ±1:09	0:23 ±1:07	p = 0.987
Examination duration, min	12:53 ±5:51	13:35 ±6:05	p = 0.358
Dosage of Atomizer only, mg	100 ±17	107 ±23	p = 0.007
Use of Rescue Anetshesia—**n (%)**	10 (7)	4 (4)	p = 0.407*
Dosage Lidocaine nasopharyngeal, mg	140 ±33	142 ±27	p = 0.570
Dosage Lidocaine endobronchial, mg	218 ±50	234 ±23	p = 0.003
Dosage Lidocaine Total, mg	359 ±62	376 ±38	p = 0.009
Dosage Lidocaine Total, mg/kg bodyweight	6.1 ±1.7	6.2 ±1.5	p = 0.551
Physician Skill Level—**n (%)**			
** - Junior Resident**	48 (33)	46 (44)	-*
** - Senior Resident**	36 (25)	21 (20)	
** - Fellow**	63 (43)	38 (36)	
Nostril intubation—**n (%)**			
** - Right**	85 (58)	55 (52)	p = 0.441*
** - Left**	62 (42)	50 (48)	
Device Size—**n (%)**			
** - Q and P (5mm)**	74 (50)	39 (37)	p = 0.041*
** - T and TQ (6mm)**	73 (50)	66 (63)	
Transbronchial biopsy performed—**n (%)**	66 (45)	58 (55)	p = 0.125*
Biopsies obtained	4.4 ±0.7	4.5 ±0.7	p = 0.484
Broncho-alveolar Lavage performed—**n (%)**	136 (93)	103 (98)	p = 0.080*
BAL recovery, %	45 ±13	44 ±12	p = 0.769
Indication—**n (%)**			
** - surveillance**	77 (52)	61 (58)	-*
** - indication**	11 (8)	2 (2)	
** - control**	59 (40)	42 (40)	

All numeric variables are shown as mean; Plus–minus values are means ±SD; All categorial variables are shown as N (%); The P value was calculated with the use of Student’s T-Test.

*The P value was calculated with the use of Chi² test if estimable

**Table 3:** Results of patients (n = 80) with returning visits (n = 160).

Characteristics	ConventionalNebulizer	DIMAD, LMA®	p-value
Patient satisfaction			
** - Efficacy**	6.8 ±2.4	7.4 ±2.3	p = 0.115
** - Side Effects**	6.5 ±2.6	7.1 ±2.4	p = 0.168
Physician satisfaction			
** - Efficacy**	6.8 ±2.5	8.1 ±2.2	p = 0.001
** - Side Effects**	6.4 ±3.0	8.3 ±2.2	p < = 0.001
Assistant satisfaction			
** - Efficacy**	6.1 ±2.5	6.8 ±2.5	p = 0.112
** - Side Effects**	6.5 ±2.5	6.7 ±2.7	p = 0.624
Improved efficacy in comparison to previous bronchoscopies—**n (%)**	18 (23)	43 (54)	p < = 0.001*
Improved effects in comparison to previous bronchoscopies—**n (%)**	15 (19)	43 (54)	p < = 0.001*
Time to investigation, min	2:07 ±0:50	2:02 ±0:33	p = 0.471
Time to nose passage, min	0:28 ±0:40	0:41 ±1:25	p = 0.211
Examination duration, min	14:24 ±6:24	15:38 ±7:16	p = 0.255
Dosage of Atomizer only, mg	100 ±18	106 ±23	p = 0.061
Use of Rescue Anesthesia—**n (%)**	8 (10)	3 (4)	p = 0.105*
Dosage Lidocaine Nasal, mg	140 ±36	146 ±37	p = 0.304
Dosage Lidocaine Endobronchial, mg	216 ±53	228 ±36	p = 0.095
Dosage Lidocain Total, mg	356 ±68	374 ±60	p = 0.079
Dosage Lidocaine Total, mg/kg bodyweight	6.2 ±1.9	5.8 ±1.6	p = 0.169
Physician Skill Level—**n (%)**			
**Junior Resident**	21 (26)	22 (28)	-*
**Senior Resident**	17 (21)	15 (19)	
**Fellow**	42 (53)	43 (54)	
Nostril intubation—**n (%)**			
**Right**	39 (49)	46 (57)	p = 0.171*
**Left**	41 (51)	34 (43)	
Device outer diameter (OD)			
OD 4.9 to 5.1 mm	31 (39)	28 (35)	p = 0.372*
OD 6.0 to 6.2 mm	49 (61)	52 (65)	
transbronchial biopsy performed, n(%)	43 (54)	39 (49)	p = 0.318*
Number of biopsies obtained	4.4 ±0.7	4.6 ±0.6	p = 0.080
Broncho-alveolar lavage performed, n(%)	72 (90)	77 (96)	p = 0.105*
BAL Recovery	55 ±14	53 ±15	p = 0.401
Indication—**n (%)**			
**surveillance**	30 (38)	27 (34)	-*
**indication**	13 (16)	11 (14)	
**control**	37 (46)	42 (53)	

All numeric variables are shown as mean; Plus–minus values are means ±SD; All categorial variables are shown as N (%); The P value was calculated with the use of Student’s T-Test.

*The P value was calculated with the use of Chi² test if estimable

**Table 4:** Safety (Complications and Side Effects).

	Patients first visit			Patient with returning visits		
Characteristics	Conventional Nebulizer (N = 147)	DIMAD, LMA® (N = 105)	p-value	Conventional Nebulizer (N = 80)	DIMAD, LMA® (N = 80)	p-value
**- Patient rated side effects -**						
Coughing–**n (%)**						
** - None**	37 (25)	24 (23)	0.856	21 (26)	30 (38)	0.311
** - Some**	97 (66)	70 (67)		47 (59)	40 (50)	
** - Strong**	13 (9)	11 (11)		12 (15)	10 (13)	
Gagging—**n (%)**						
** - None**	37 (25)	34 (32)	0.940	23 (29)	29 (36)	0.161
** - Some**	76 (52)	52 (50)		42 (53)	44 (55)	
** - Strong**	26 (18)	19 (18)		15 (19)	7 (9)	
Unpleasant taste–**n (%)**						
** - None**	37 (25)	30 (29)	0.300	21 (26)	23 (29)	0.189
** - Some**	68 (46)	30 (51)		33 (41)	41 (51)	
** - Strong**	52 (29)	21 (20)		26 (33)	16 (20)	
Nausea–**n (%)**						
** - None**	124 (84)	92 (88)	0.438	64 (80)	67 (84)	0.349
** - Some**	18 (12)	12 (11)		14 (18)	13 (16)	
** - Strong**	5 (3)	1 (1)		2 (3)	0 (0)	
Burning sensation–**n (%)**						
** - None**	79 (54)	64 (61)	0.283	38 (48)	54 (68)	0.017
** - Some**	56 (38)	37 (35)		36 (45)	19 (24)	
** - Strong**	12 (8)	4 (4)		6 (8)	7 (9)	
Pain–**n (%)**						
** - None**	96 (65)	76 (72)	0.477	46 (58)	57 (71)	0.126
** - Some**	45 (31)	25 (24)		32 (40)	20 (25)	
** - Strong**	6 (4)	4 (4)		2 (3)	3 (4)	
**- Physician rated complications -**						
Epistaxis—**n (%)**						
** - Yes**	3 (2)	1 (1)	0.496	1 (1)	2 (3)	0.56
** - No**	144 (98)	104 (99)		79 (99)	78 (98)	
Minor bleeding—**n (%)**						
** - Yes**	44 (30)	31 (30)	0.944	32 (40)	25 (31)	0.161
** - No**	103 (70)	74 (71)		48 (60)	55 (69)	
Vomiting—**n (%)**						
** - Yes**	1 (1)	0 (0)	-	1 (1)	2 (3)	0.56
** - No**	146 (99)	105 (100)		79 (99)	78 (98)	
Laryngospasm—**n (%)**						
** - Yes**	6 (4)	3 (3)	-	5 (6)	3 (4)	0.47
** - No**	141 (96)	102 (97)		75 (94)	77 (96)	
Moderate bleeding—**n (%)**						
** - Yes**	17 (12)	8 (8)	0.302	5 (6)	6 (8)	0.500
** - No**	130 (89)	97 (92)		75 (94)	74 (93)	
Desaturation—**n (%)**						
** - Yes**	1 (1)	0 (0)	-	0 (0)	1 (1)	-
** - No**	146 (99)	105 (100)		80 (100)	79 (99)	

Side effects evaluated by patients after the bronchoscopy was conducted; All categorial variables are shown as N (%);The P value was calculated with the use of Chi² test if estimable

Total dose of lidocaine and nasal dose was slightly higher with the disposable atomizer for the patients with one visit. Nasal dose was not different for returning patients (Tables 2 and 3).

### Cost calculations

The original costs for Conventional reusable nasal Atomizer (CRNA) are 0,16€ per procedure, based on mean durability of 505 procedures, along with a further 0,08 € per procedure for the rubber bulb (mean durability 116 procedures). The processing costs for CRNA are 3,84€ per procedure, resulting in a total cost of 4,08€ per procedure. The disposable Intranasal Mucosal Atomization Device (DIMAD) currently cost 3,70€ each. There was no difference in time between administering local anesthesia and beginning the bronchoscopy or in the time required to negotiate the nasal passage (Tables 2 and 3).

## Discussion

In this prospective cross-over trial we compared two devices for topical nasal anesthesia during flexible bronchoscopy (FB) in an outpatient setting. We could demonstrate, that topical nasal anesthesia with 2% lidocaine given over a single-use disposable intranasal mucosal atomization (DIMAD) device was non-inferior to conventional reusable nasal atomizers in unsedated patients.

Although most guidelines recommend FB under intravenous sedation, centers with large volumes of outpatient bronchoscopy, tend to avoid general anaesthesia, due to limited observation and physician resources [4, 9, 10]. Topical upper airway anesthesia with lidocaine achieved sufficient patient comfort with effective cough reflex suppression to facilitate safe and effective bronchoscopy [22]. The British Thoracic Society recommends topical upper airway anesthesia with 2% lidocaine gel (nasal), and 1–4% lidocaine solution whilst employing a ‘spray-as-you-go’ technique [9, 22]. In our experience sole use of 2% lidocaine gel is inadequate when using large bore bronchscopes (>5 mm) in the absence of sedation.

Middleton and colleagues assessed patient comfort in transnasal bronchoscopy, concluding that any regimen appeared acceptable as long as viscous lidocaine was included [23]. To date, no published trials have investigated using low-dose (2%) lidocaine gel. Lidocaine spray might be advantegeous to reach the posterior nasopharynx in comparison to lidocaine gel.

*S*ince the introduction of transnasal flexible bronscocopy, various techniques of nasal anesthesia have been developed locally, and passed on to successive generations of bronchoscopists based on experience [24]. Few studies have investigated nasal anesthesia during transnasal FB. Some small studies reported patient preference for nasal lidocaine gel rather than spray [5, 6, 23, 25]. Webb *et al*. reported in 1989 regarding patient preference for 2% lidocaine gel compared to 10% spray [5]. All patients had however received additional sedatives and analgetics. Patient comfort was assessed by post-interventional questionnaire, introducing a risk of bias from the analgosedation.

Another study reported 10% lidocaine spray as the least pleasant medication, with study participants preferring lower concentrations of lidocaine spray or topical gel application [25]. Unfortunately, in this study no complete bronchoscopies were performed, with the scope being passed through the nose only as simulation.

Our data demonstrate that nasal anesthesia with DIMAD is safe with non-inferior patient comfort and satisfaction (Tables 2 and 3). There was no difference in the time interval between application of local anesthesia to starting bronchoscopy, or in the time taken to negotiate the nasal passage between devices. The DIMAD proved easier to use and may allow future administration by nursing staff, resulting in time savings for the physician.

To our knowledge, there are no studies in the past years, which investigated other techniques for nasal anesthesia as part of topical anesthesia for FB.

The main limitations of the study are the single center, non-randomized design. Neither the physician nor assistants were blinded to the study treatment. This might be a strong bias. The most differences were noticed by the non-blinded staff, not the patients.

Only patients after lung transplantation were included. On one hand these patients are well experienced with the procedure and non-sedation but on the other hand conclusions in this selective population may limit extrapolation of the findings to a certain degree.

In conclusion, topical nasal anesthesia with low-dose lidocaine spray proofed to be safe. A disposable intranasal mucosal atomization device (DIMAD) for mucosal nasal anesthesia was non-inferior in terms of efficacy. The device was preferred by physicians and was cost effective.

## Supporting Information

S1 CONSORT Checklist (DOC)

S1 Questionnaire (Original Version). (DOCX)

S2 Questionnaire (English Version). (DOCX)

S1 Study Protocol (DOC)

## Abbreviations

BAL: bronchoalveolar lavage
DIMAD: disposable intranasal mucosal atomization device
IPF: idiopathic pulmonary fibrosis
LTx: lung transplantation
RNA: reusable nasal atomizer
