# Supplementary material for: Topical Nasal Anesthesia in Flexible Bronchoscopy – A Cross-Over Comparison between Two Devices
Source: PLoS One. 2016 Mar 15;11(3):e0150905. doi: 10.1371/journal.pone.0150905 (PMC4792394; doi:10.1371/journal.pone.0150905)
Supplement: S1 Questionnaire — (DOCX) [file pone.0150905.s002.docx]

# 01 Sind bei Ihnen schon früher Bronchoskopien durchgeführt worden?

Ja Nein

# 02 Sind bei Ihnen schon früher Bronchoskopien durch die Nase durchgeführt worden?

Ja Nein

# 03 Wenn ja, war die Bronchoskopie durch die Nase früher schwierig?

Ja Nein

# 04 Wie beurteilen Sie die Wirksamkeit des Betäubungssprays im Nasen-Rachenraum bei der heutigen Bronchoskopie insgesamt?

| < Sehr schlecht |  | Sehr Gut > |
| --- | --- | --- |

# 05 Wie stark empfanden Sie die Nebenwirkungen des Betäubungssprays im Nasen-Rachenraum bei der heutigen Bronchoskopie insgesamt?

| < Sehr stark |  | Sehr gering > |
| --- | --- | --- |

# 06 Welche Nebenwirkungen hatten Sie durch Verabreichung des Betäubungssprays im Nasen-Rachenraum?

## A Husten

Stark Etwas Gar nicht

## B Würgereiz

Stark Etwas Gar nicht

## C Schlechter Geschmack

Stark Etwas Gar nicht

## D Übelkeit

Stark Etwas Gar nicht

## E Brennen

Stark Etwas Gar nicht

## F Schmerzen

Stark Etwas Gar nicht

# 07 Wie war die Wirksamkeit des Betäubungssprays im Nasen-Rachenraum im Vergleich zu früheren Bronchoskopien?

viel besser etwas besser unverändert etwas schlechter viel schlechter

# 08 Wie beurteilen Sie die Nebenwirkungen des Betäubungssprays im Nasen-Rachenraum im Vergleich zu früheren Bronchoskopien?

Viel besser etwas besser unverändert etwas schlechter viel schlechter
