# Supplementary material for: Topical Nasal Anesthesia in Flexible Bronchoscopy – A Cross-Over Comparison between Two Devices
Source: PLoS One. 2016 Mar 15;11(3):e0150905. doi: 10.1371/journal.pone.0150905 (PMC4792394; doi:10.1371/journal.pone.0150905)
Supplement: S2 Questionnaire — (DOCX) [file pone.0150905.s003.docx]

# 01 Have bronchoscopies been carried out at you earlier?

Yes No

# 02 Have bronchoscopies been carried out at you earlier through the nose?

Yes No

# 03 If Yes, were there difficulties conducting the bronchoscopy through the nose?

Yes No

# 04 How do you assess the effectiveness of the anesthetic sprays in the nasopharynx with the today's bronchoscopy total?

| < much worse |  | very good > |
| --- | --- | --- |

# 05 How strong you felt the side effects of anesthetic sprays in the nasopharynx with the today's Bronchoscopy total?

| < very strong |  | non existant > |
| --- | --- | --- |

# 06 What side effects did you have by the administration of the anesthetic sprays in the nasopharynx?

## A Cough

strong some none

## B Gagging

strong some none

## C Unpleasant Taste

strong some none

## D Nausea

strong some none

## E Burning Sensation

strong some none

## F Pain

strong some none

# 07 How was the efficacy of anesthetic sprays in the nasopharynx in comparison to previous bronchoscopies?

much better slightly better unchanged somewhat worse much worse

# 08 How do you assess the side effects of anesthetic sprays in the nasopharynx in comparison to previous bronchoscopies?

much better slightly better unchanged somewhat worse much worse
