# Supplementary material for: Topical Nasal Anesthesia in Flexible Bronchoscopy – A Cross-Over Comparison between Two Devices
Source: PLoS One. 2016 Mar 15;11(3):e0150905. doi: 10.1371/journal.pone.0150905 (PMC4792394; doi:10.1371/journal.pone.0150905)
Supplement: S1 Study Protocol — (DOC) [file pone.0150905.s004.doc]

# Outline Clinical Trials Application (6 pages max.)

**funding measure: core funding**

1. STUDY SYNOPSIS

| **APPLICANT/COORDINATING INVESTIGATOR** | PD Dr. med. Thomas Fühner  PD. Dr. med Jens Gottlieb  Medizinische Hochschule Hannover,  Department of Respiratory Medicine,  Carl-Neuberg Str. 1  30265 Hannover  Phone-Nr.: 0511-532-4601  Fax-Nr.: 0511-532-8094  Gottlieb.jens@mh-hannover.de  Fuehner.thomas@mh-hannover.de |
| --- | --- |
| **TITLE OF STUDY** | Topical nasopharyngeal anesthesia in outpatient bronchoscopy |
| **CONDITION/TOPIC** | **Topical anesthesia / bronchoscopy** |
| **OBJECTIVE(S)** | **Patient satisfaction** |
| **INTERVENTION (S)** | Experimental intervention: topical anesthesia.  Control intervention: none  Duration of intervention per patient/subject:  3 months |
| **KEY INCLUSION AND EXCLUSION CRITERIA** | Key inclusion criteria:   - patients after lung transplantation (single, double or combined) - Informed consent - outpatient bronchoscopy   Key exclusion criteria:   - Oxygen requirement at rest - Need for peri-interventional sedation - limited German language skills or other reasons which might impair patient communication or computer handling |
| **OUTCOME(S)** | Primary efficacy endpoint:   - patient self-rated nasopharyngeal symptoms after topical nasopharyngeal anesthesia   Key secondary endpoint(s):   - time of delivery of topical nasopharyngeal anesthesia - costs of topical nasopharyngeal anesthesia - dose of lidocaine for topical nasopharyngeal anesthesia - health care giver rated nasopharyngeal symptoms after topical nasopharyngeal anesthesia - side effects of topical nasopharyngeal anesthesia - need for rescue medication for topical nasopharyngeal anesthesia - Assessment of safety: - side effects of topical nasopharyngeal anesthesia   . |
| **STUDY TYPE** | *Prospective observational study* |
| **STATISTICAL ANALYSIS** | Efficacy:   - In the middle of the study period topical anesthesia will be changed from the Laryngeal atomizer to the Intranasal Mucosal Atomization Device. - The primary aim of this study is to compare a simplified single-use product (LMA® MAD Nasal™ Intranasal Mucosal Atomization Device, Teleflex medical, Kernen Germany) for topical nasopharyngeal anesthesia in comparison to a multi-use device (Laryngeal atomizer, Karl Storz, Tuttlingen, Germany in terms of patient self-rated nasopharyngeal symptoms. Patients included in the first half of the study (topical anesthesia with the Laryngeal atomizer) and with need for another bronchoscopy in the second half of the study (topical anesthesia with the Intranasal Mucosal Atomization Device) will be asked to repeat the questionnaire after the second bronchoscopy to allow in-patient comparison.   Description of the primary efficacy analysis and population:  The primary endpoint of patient self-rated anesthesia by visual analogue scale after topical nasopharyngeal anesthesia for bronchoscopy. |
| **SAMPLE SIZE** | To be assessed for eligibility (n = 300)  To be allocated to trial (n = 200)  To be analysed (n = 200) |
| **TRIAL DURATION** | First patient/subject in to last patient/subject out: Q3/2014  Duration of the entire trial: 3 months |
| **PARTICIPATING CENTERS** | Hannover Medical School  Dpt. Respiratory Medicine OE 6870  Carl-Neuberg Str. 1  30625 Hannover |
| **PREVIOUS DFG/BMBF PROJECT NUMBER** | none |
